# Efficiency of Magnetostatic Protection Using Nanostructured Permalloy Shielding Coatings Depending on Their Microstructure

**DOI:** 10.3390/nano11030634

**Published:** 2021-03-04

**Authors:** Tatiana Zubar, Sergey Grabchikov, Anna Kotelnikova, Egor Kaniukov, Maksim Kutuzau, Karin Leistner, Kornelius Nielsch, Tatiana Vershinina, Daria Tishkevich, Oleg Kanafyev, Artem Kozlovskiy, Maxim Zdorovets, Valery Fedosyuk, Alex Trukhanov

**Affiliations:** 1 Laboratory of Magnetic Films Physics, Scientific-Practical Materials Research Centre of National Academy of Sciences of Belarus, 220072 Minsk, Belarus; gss@physics.by (S.G.); anna.kotelnikova.98@mail.ru (A.K.); dashachushkova@gmail.com (D.T.); olegkan96@mail.ru (O.K.); fedosyuk@physics.by (V.F.); truhanov86@mail.ru (A.T.); 2 Laboratory of Single Crystal Growth, South Ural State University, 454080 Chelyabinsk, Russia; 3 Department of Technology of Electronics Materials, National University of Science and Technology MISiS, 119049 Moscow, Russia; ka.egor@mail.ru; 4 Leibniz IFW Dresden, Helmholtzstrasse 20, 01069 Dresden, Germany; m.kutuzau@ifw-dresden.de (M.K.); k.nielsch@ifw-dresden.de (K.N.); 5 Institute of Material Science, TU Dresden, 01062 Dresden, Germany; 6 Joint Institute for Nuclear Research, 141980 Dubna, Russia; vershinina@nf.jinr.ru; 7 The Institute of Nuclear Physics, Almaty 050032, Kazakhstan; artem88sddt@mail.ru (A.K.); mzdorovets@gmail.com (M.Z.); 8 Engineering Profile Laboratory, L.N. Gumilyov Eurasian National University, Nur-Sultan 010008, Kazakhstan; 9 Department of Intelligent Information Technologies, The Ural Federal University, 620002 Yekaterinburg, Russia

**Keywords:** permalloy, pulsed electrodeposition, nanostructured coating, microstructure, magnetostatic shielding

## Abstract

The effect of microstructure on the efficiency of shielding or shunting of the magnetic flux by permalloy shields was investigated in the present work. For this purpose, the FeNi shielding coatings with different grain structures were obtained using stationary and pulsed electrodeposition. The coatings’ composition, crystal structure, surface microstructure, magnetic domain structure, and shielding efficiency were studied. It has been shown that coatings with 0.2–0.6 µm grains have a disordered domain structure. Consequently, a higher value of the shielding efficiency was achieved, but the working range was too limited. The reason for this is probably the hindered movement of the domain boundaries. Samples with nanosized grains have an ordered two-domain magnetic structure with a permissible partial transition to a superparamagnetic state in regions with a grain size of less than 100 nm. The ordered magnetic structure, the small size of the domain, and the coexistence of ferromagnetic and superparamagnetic regions, although they reduce the maximum value of the shielding efficiency, significantly expand the working range in the nanostructured permalloy shielding coatings. As a result, a dependence between the grain and domain structure and the efficiency of magnetostatic shielding was found.

## 1. Introduction

The issue of creating effective electromagnetic shields for protection from both magnetostatic fields and electromagnetic radiation is critically essential nowadays [1,2,3,4,5,6]. The level of the electromagnetic background, caused by the rapid development of electrical, radio-electronic, transport, information, and military technology, has significantly increased, the range of used frequencies of electromagnetic radiation has expanded, and their amplitude has increased. On the other hand, the development of radio-electronic and information technology, research, and intelligent complexes is moving toward minimizing the mass and dimensions as well as increasing the density of their arrangement. All this significantly complicates the problem of the formation of an electromagnetic environment that provides the regular functioning and electromagnetic compatibility of electrical and radio-electronic complexes, which requires the development of new specialized materials that ensure reliable and stable functioning under conditions of intentional and unintentional electromagnetic influences [7,8,9,10,11,12]. Beyond that, the importance of the problem task of electromagnetic protection of biological objects from the effects of various kinds of electromagnetic radiation and permanent magnetic field is becoming increasingly obvious [13,14,15,16,17,18].

Analytical calculations assume that magnetic materials with the highest value of magnetic permeability provide better protection. Magnetostatic shielding usually shunts the magnetic field via a ferromagnetic material [11,19]. It essentially closes the force lines through the material with low resistance to the magnetic flux. The R_m_ resistance value in the magnetic flux uses a shield with *μ_r_* magnetic permeability, *l* average length of the magnetic induction lines through the material, and *S* cross-section in a perpendicular direction to the magnetic flux:

R_m_ = *l*/*μ_r_S*. (1)

For magnetostatic protection, the shielding efficiently (SE_ms_) in first principle could be described by equation:

SE_ms_ = 1 + *μ_r_d*/*D* (2)

where *μ_r_*—relative magnetic permeability; *d*—thickness of the shield; and *D*—diameter of the cylindrical or spherical shield [11,12]. However, earlier, it was noted [19,20] that the theoretical equations cannot accurately describe the efficiency of the shield; it only characterizes the dependence of the SE on certain parameters. It should be pointed out that growing the shield thickness is always a faultless option in terms of enhancing SE. However, the large size and weight of the protection shield is often unacceptable from a design point of view. Therefore, it is necessary to search for other approaches to increasing the SE.

The most commonly used material for creating magnetostatic and electromagnetic shields is soft magnetic alloy of Ni and Fe or permalloy. Ni-rich permalloy has high permeability, low coercivity, and small magnetic anisotropy [11,17,19,21,22,23,24,25,26,27,28,29]. Electrodeposited coatings are promising due to the high economic viability of the electrodeposition process [29,30,31,32]. The ability to deposit a protection coating on the substrates with a complex shape should also be noted among the main advantages of electrodeposition. This is important for using electromagnetic shields, when it is necessary to cover part of the package or complex shape part.

In this work, we studied the effect of permalloy structure on the efficiency of shielding (or shunting) of the magnetic flux. No studies were found on this topic in the earlier literature. We assumed that the features of the process of magnetization of ferromagnetic permalloy in a constant magnetic field can have a significant effect on the shielding efficiency. One of the simplest ways to control the magnetization stages of ferromagnetic materials (changing the size of domains, absorbing domains, and ordering the vectors of magnetic moments over the field) is to change the structure of the material. Therefore, we formed FeNi shields with different grain structures and studied their magnetic domain structure using magnetic force microscopy. As a result, a relationship between the domain and grain structure and the efficiency of magnetostatic shielding was found. In addition, the fundamental possibility of controlling the width of the working range and the value of the efficiency of the magnetostatic permalloy shield was demonstrated.

## 2. Materials and Methods

Electrodeposition was used to obtain FeNi shielding coatings. The substrates for the electrodeposition were aluminum alloy cylinders. The cylinder’s length was 100 mm and its external diameter was 30 mm. Aluminum alloy was chosen as more suitable because it has low weight, high mechanical and corrosive characteristics, as well as high electrical conductivity required for electrodeposition. The disadvantage is poor adhesion of Al-alloy to coating. For this reason, complex surface preparation was carried out. At the first stage, the surface was cleaned from organic contaminants using trichloroethylene and a mixture of calcium oxide (90 wt %) and magnesium oxide. Then, degreasing and cleaning was carried out in a solution of H_25_Na_2_O_16_P—40g/L, Na_2_CO_3_—40 g/L, and NaOH—40 g/L at a temperature of 70 °C. After that, the surface was chemically etched in the nitrogen (300 mL/L) and hydrofluoric (100 mL/L) acid solution to remove sludge. The obligatory stage of Al surface preparation is the creation of a thin adhesive layer of Zn. To obtain a uniform and compact layer, zinc is applied chemically in two stages: chemical coating in solution (ZnSO_4_—250 g/L, NaOH—450 g/L, and KNaC_4_H_4_O_6_—7 g/L), removal of the upper loose zinc layer in 50% solution of HNO_3_, and the deposition of a second Zn layer. The last stage of the surface preparation is the electrolytic deposition of a copper sublayer with a thickness of 3 μm. The solutions for Cu deposition contains cuprous sulfate—30 g/L, potassium phosphate—140 g/L, sodium phosphate trisubstituted dodecahydrate—90 g/L, and sodium potassium tartrate—20 g/L. The Cu-electrolyte temperature was 35 °C, current density was 8 mA/cm^2^, and pH level was 8.5. The cupper deposition rate with such parameters is 9 μm/h.

**Figure 1:**
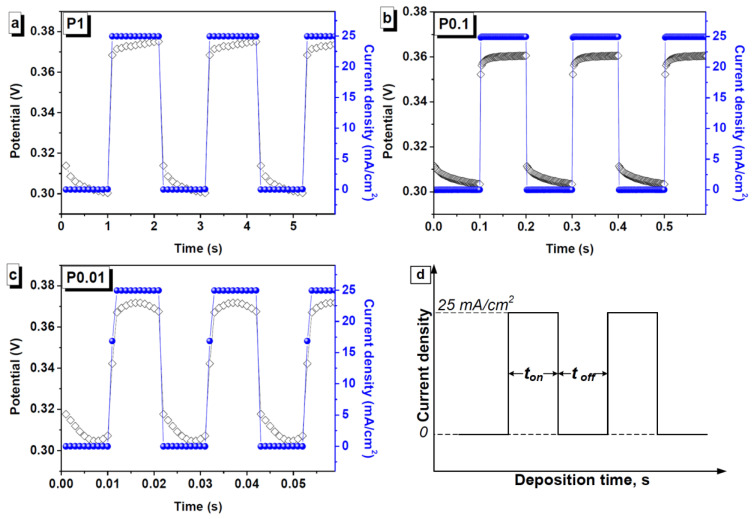
Change in current density and potential during pulsed deposition of the (**a**) P1, (**b**) P0.1, and (**c**) P0.01 shields, as well as (**d**) a schematic representation of pulsed deposition.

The FeNi coatings were deposited from a complex electrolyte containing NiSO_4_ 7H_2_O—250 g/L, NiCl_2_ 6H_2_O—20 g/L, H_3_BO_3_—25 g/L, MgSO_4_ 7H_2_O—110 g/L, FeSO_4_ 7H_2_O—35 g/L, D (+) Glucose—85 g/L, HC_6_H_7_O_6_—3 g/L, and additive NiB1—3 g/L. The temperature was kept at 35°C and pH was kept at 2.0. The current density was 35 mA/cm^2^. Under these conditions, the deposition rate was 35 μm/h. The described electrodeposition technology makes it possible to obtain permalloy coatings with a nickel and iron ratio of 50–50 at % and satisfactory mechanical and aesthetic characteristics. A description of the technological features of obtaining samples of shields in direct current and pulse modes [33,34] is presented in Table 1. Sample P0 was obtained at direct current for 85 min. Samples P1, P0.1, and P0.01 were obtained in pulsed modes with pulse durations of 1, 0.1, and 0.01 s, respectively. The pause time was equal to the pulse time. Figure 1 shows graphs of changes in current density and potential for pulse modes. 

AZtecLive Advanced with Ultim Max 40 (Oxford Instruments, Bognor Regis, UK) investigated the chemical composition using energy-dispersive X-ray spectroscopy. The crystal structure investigation was carried out by X-ray structural analysis on an EMPYREAN (PANalytical, Malvern Instruments, Malvern, UK) powder diffractometer using Cu-Kα radiation in the Bragg–Brentano geometry focusing in the angle range 2θ = 40–100°. The sizes of the coherent scattering regions (CSR) were estimated using the Williamson–Hall method for all peaks from the fcc solid solution. The surface microstructure was studied using the scanning electron microscope Zeiss EVO 10 (Zeiss, Oberkochen, Germany) and atomic force microscope (AFM) Bruker Demension Icon (Bruker Corporation, Billerica, MA, USA). Magnetic force microscopy (MFM) imaging was performed in a dual-scan mode with Bruker Demension Icon microscore. The first scan pass was used for the morphological imaging; the second pass was operated at constant height from the surface (100 nm). The advantage of the applied MFM method, in addition to its large lateral resolution, is that long-range magnetic interactions can be virtually excluded [35]. A silicon probe with CoCr thin coating was used. The probe coercivity was about 400 Oe and the magnetic moment was about 10^−13^ EMU. The AFM tip diameter was about 20 nm. The lateral resolution of AFM and MFM images can be higher than the tip’s curvature due to non-contact scanning and the regularity of the surface [36,37,38,39,40]. The resonant frequency during scanning was 80 kHz, and the force constant = 2.8 N/m [41]. The magnetic tip was magnetized by an external magnet and tested with a magnetic calibration grid prior to measurements.

**Figure 2:**
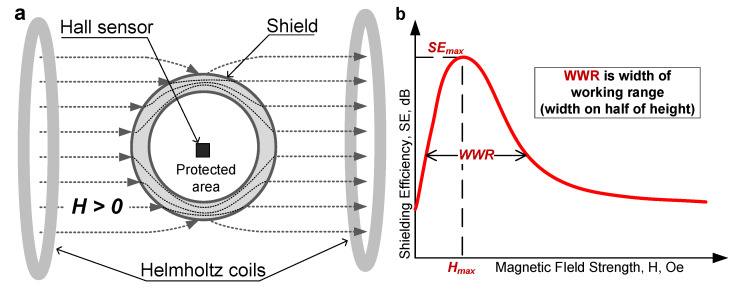
Schematic representation of the shielding efficiency measurement: (**a**) installation for shielding efficiency (SE) measurement of the cylindrical shields and (**b**) schematic representation of graph of the change in SE.

For shielding efficiency (SE) measurements, the cylindrical sample was placed in a uniform magnetic field created by a pair of Helmholtz coils (Figure 2a). The calculation of SE is based on measurements of the Hall potential in the protected region. The SE was determined as the ratio magnetic field strength without H_ext_ and with an H_int_ shield using Equations (3) and (4).

SE= H_ext_/H_int_ (3)

SE=20 lg(H_ext_/H_int_) [in dB]. (4)

The parameters SE_max_, H_max_, and WWR (width of working range) were used to quantify the shielding efficiency of the samples. Figure 2b shows schematically how these parameters were determined. The maximum value of the shielding efficiency SE_max_ and the strength of the magnetic field H_max_, which corresponds to this maximum, are determined as the extremum of the function SE(H). WWR is the width of the peak of the experimental graph at half height. The WWR value characterizes the width of the shield’s working range.

## 3. Results and Discussion

**Figure 3:**
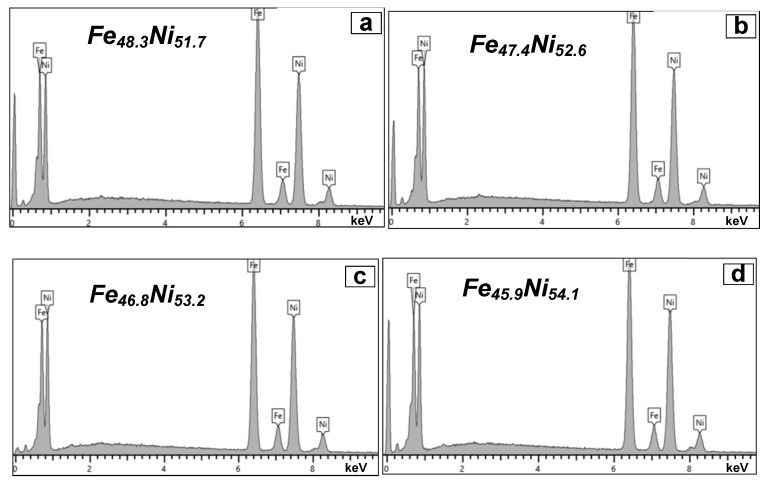
Nominal chemical composition of permalloy shielding coating: (**a**) P0, (**b**) P1, (**c**) P0.1, and (**d**) P0.01.

The results of the analysis by energy-dispersive X-ray spectroscopy are presented in the form of spectra in Figure 3. The percentage of nickel and iron obtained from the analysis of the spectra is presented as a caption on the corresponding spectra. There is a slight (less than 1 at %) but stable decrease in the concentration of Fe with a decrease in the pulse duration. The reason of the Fe content decrease is a significant change in technological parameters: the transition from stationary to pulsed deposition mode and a sharp decrease in the pulse duration. A change in technological parameters can lead to a shift in the redox reaction

Me^2+^ + 2e → Me^0^ (5)

Different electrodeposition parameters can influence the Fe/Ni ratio in opposite directions. For example, an increase in the electrolyte temperature intensifies the oxidation of Fe ions (transition from Fe^2+^ to Fe^3+^). The iron content in the film decreases as a result of oxidation [42,43]. It was previously shown by many authors that during the deposition of binary and ternary alloys [44,45,46], a composition gradient is observed from the substrate to the coating surface (for the FeNi alloys, a decrease in the nickel content is observed). It is also known that iron is predominantly deposited on the irregularities and boundaries of the substrate and grains in electrodeposited alloys. The reason for the high concentration of iron in “ridge” is the higher current density in this area [47]. It is also reported that a high substrate roughness leads to a decrease in the iron content, since the current density decreases in terms of the actual surface area (taking into account the roughness). Thus, the chemical composition is determined by the competition of many phenomena. A decrease in the pulse time contributed to a decrease in the Fe content in the considered case. This is probably due to the structure of the coating surface. It will be shown below that the grain size decreases with decreasing pulse duration, which is natural and widely studied [47,48]. This led to an increase in the roughness and a decrease in the current density during the formation of the coating.

**Figure 4:**
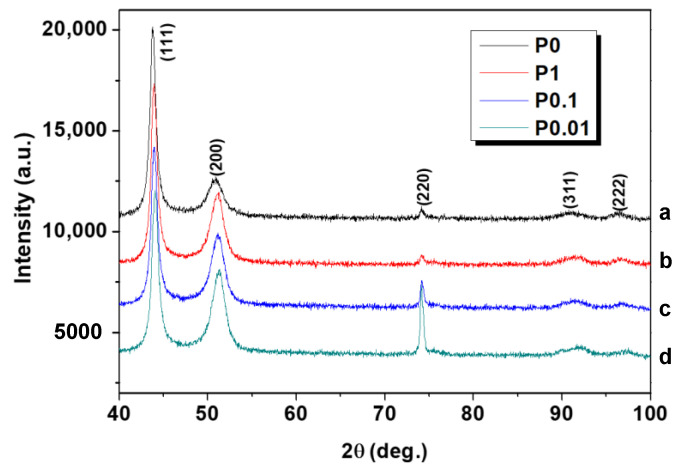
XRD patterns of FeNi shielding coatings: (a) P0, (b) P1, (c) P0.1, and (d) P0.01.

Figure 4 and Table 2 demonstrate results of XRD investigations of the FeNi shielding coating obtained by the different modes. The number of well-distinguished peaks can be observed on the XRD patterns. The most intense and characteristic peaks are 43–44 deg., 50–51 deg. (corresponding to the atomic plane (111)), and 74 deg. (the atomic plane (220)). A decrease in the width of the peaks was observed as the pulse duration was shortened. The evaluations carried out by the Williamson–Hall method showed that the main factor causing the change in the width of the X-ray peaks is the size of the CSR. Table 2 shows that the CSR was 3.9 and 3.3 nm for the samples obtained in the P0 and P1 modes. Then, an increase in CSR to 5.1 and 5.0 nm is observed for samples P0.1 and P0.01, respectively. It was noted that coatings deposited in pulsed modes have a common feature: the X-ray diffraction patterns show the change in the ratio of the peaks (111) and (200) when switching to pulsed modes. If we take nickel as the starting point, then the ratio of the integral intensities (111) and (200) for a completely disordered state is 46%. As can be seen in Table 1, the peak of the crystallographic plane (200) for sample P0 has a ratio I_(111)_/I_(200)_, which is close to the intensity of the completely isotropic material (57%). The most intense peak (111) of the fcc lattice is taken as 100%. The contribution of the peak (200) increases and the ratio I_(111)_/I_(200)_ reaches 84, 91, and 95% for P1, P0.1, and P0.01 coatings, respectively. It should also be noted that a decrease in the pulse duration leads to an increase in the detected effect. The unit cell parameter (a) and cell volume (V) decrease nonlinearly from 3.578 to 3.567 Å and from 45.80 to 45.38 Å^3^ with transition from stationary to pulsed electrodeposition and with the pulse duration decreasing. The reason for the compression of the crystal lattice can be two factors: (1) surface compression of grains with a decrease in their size [27,49,50], and (2) the effect of the chemical composition (a decrease in the Fe content with an atomic radius bigger (r_Fe_ = 0.156 nm) than that of Ni (r_Ni_ = 0.124 nm)).

**Figure 5:**
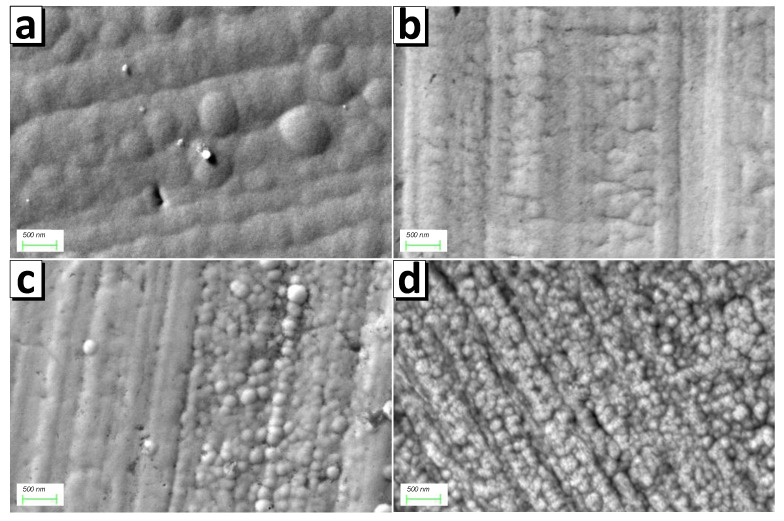
Surface microstructure of FeNi shields investigated using SEM: (**a**) P0, (**b**) P1, (**c**) P0.1, and (**d**) P0.01.

The surface microstructure of the permalloy shielding coating investigated using SEM is shown in Figure 5. As expected, the grain size decreased upon transition to pulse electrodeposition and the decrease in the pulse duration. While increasing the grain size is more energetically beneficial than the formation of a new growth nucleus, the coating grains grow continuously in stationary electrolysis [34,51]. The coating with a grain size of 500–600 nm was formed (Figure 5a) by stationary deposition. When switching to the pulse mode P1, the grains grow for 1 s. Growth stops when the current is turned off. Then, when the current is switched on again, the growth of predominantly new grains begins [52,53,54]. The size of grains formed within 1 s ranges from 150 to 300 nm (Figure 5b). A decrease in the pulse duration leads to a decrease in the grain size. Thus, the grain size of the P0.1 shield is in the range from 100 to 200 nm, and almost all grains are less than 100 nm for the P0.01 shield.

**Figure 6:**
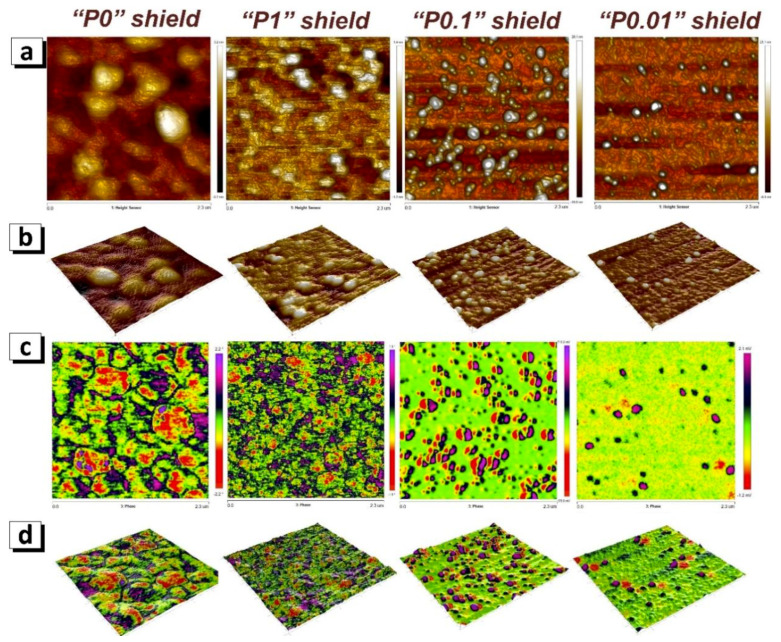
Surface microstructure of FeNi shielding coating investigated using atomic force microscope (AFM) and magnetic domain structure investigated using magnetic force microscopy (MFM, scan sizes are 2.3 μm × 2.3 μm): (**a**) 2D surface microstructure, (**b**) 3D surface microstructure, (**c**) 2D domain structure, (**d**) domain structure superimposed as a colored skin on the 3D image of the surface microstructure.

Figure 6a shows the change in the microstructure obtained using AFM. Figure 6b is a 3D rendering of the corresponding image of surface topography. The results (surface structure and grain size) are in good agreement with the SEM results (Figure 5). A number of images (Figure 6c) show the results of studying the magnetic domain structure of the surface regions corresponding to the AFM images. Comparison of Figure 6a,c allows us to draw several comments.

1. There is practically no ordered domain structure for the P0 and P1 modes. High values of magnetization (perpendicular to the coating plane in modulus) correspond to the central regions of grains, and low values correspond to grain boundaries.
2. A two-domain structure (magnetization vectors up and down) is formed within grains with size 150–200 nm) for the P0.1 coating.
3. One domain is formed in grains with a size of 100–150 nm with the magnetization vectors perpendicular to the coating plane (purple regions). Another oppositely directed domain is formed near but outside the grain to compensate (red regions). This is observed for coatings P0.1 and P0.01.
4. The magnetization vectors of grains with a size less than 100 nm lie in the plane of the coating and are magnetized uniformly or not magnetized (light green color for coatings P0.1 and P0.01).

Figure 6d is a 3D visualization of the surface topography with a contrasting image superimposed on top as a skin, demonstrating the magnetic structure. This is done to better understand the correspondence of the domain structure to the surface microstructure.

**Figure 7:**
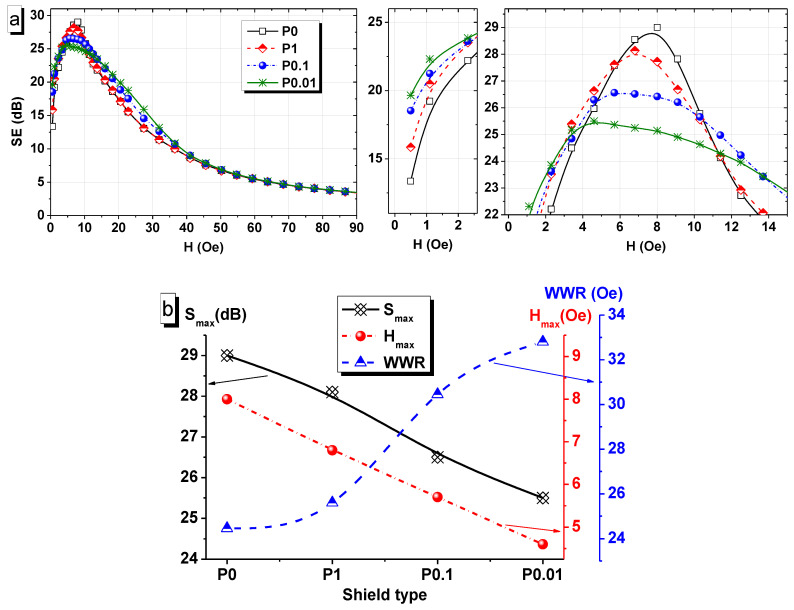
SE of FeNi shields: (**a**) dependence of SE on the strength of the applied magnetic field in the range from 0 to 90 Oe, (**b**) the maximum value of the shielding efficiency (SE_max_—black), the strength of the magnetic field that corresponds to SE_max_ (H_max_—red) and the width of the shield’s working range (WWR—blue).

Figure 7a shows the change in the SE of the cylindrical shields with permalloy coatings obtained in different modes with an increase in the magnetic field strength up to 90 Oe. Significant differences between the shields disappear in a magnetic field of more than 50 Oe; however, in the range of 0–50 Oe, all shields show different efficiency characteristics. The maximum SE (SE_max_) of the P0 shield is 20 dB, and the SE_max_ for the P0.01 shield is 25.5 dB, or 30 and 19 times, respectively (Figure 7b, black line). The maximum field value (H_max_) changes in the same way as the SE_max_; a linear decrease from 8 to 4.6 Oe is observed with transition from stationary to pulsed electrodeposition and with decreasing the pulse duration to 0.01 s (red line on Figure 7b). Nevertheless, the width of the working range or WWR (blue line on Figure 7b) of the shields increases nonlinearly for the same transition of modes from the P0 coating to the P0.01 coating. Thus, the width is 24.5, 25.6, 30.5, and 32.8 Oe for the P0, P1, P0.1, and P0.01 samples, respectively.

The behavior of the SE is directly dependent on the magnetic domain structure. It is known that the magnetization of a ferromagnetic material, such as permalloy, occurs in several stages. (1) The ferromagnetic initially is unmagnetized without the magnetic field; (2) in a small magnetic field, domains that are collinear with the field increase in size and uncollinear domains decrease in sizes; (3) collinear large domains absorb small uncollinear ones with a further increase in the field strength; and (4) all magnetization vectors are ordered according to the magnetic field.

Studies of the magnetic domain structure with MFM in a small magnetic field induced by a CoCr probe have shown that the size of a domain or a region with uniform magnetization decreases with a decrease in the grain size. The estimated magnetic field of the probe does not exceed 1 Oe. This corresponds to the starting point on the SE curves (Figure 7a). At the H = 0.5 Oe, the SE is 13.4, 15.8, 18.5, and 19.6 dB for the P0, P1, P0.1, and P0.01 shields, respectively. An increase in the magnetic field leads to an increase in the size of the domains directed collinear to the field. Previously, many experts reported that moving domain boundaries is less energy-intensive than absorption domains with each other [55,56,57,58]. Based on the MFM data, we can conclude that samples P0 and P1 are magnetized mainly due to the absorption of neighboring domains, and samples P0.1 and P0.01 are due to an increase in the size of domains during the absorption of disordered regions. In addition, the remagnetization of small domains requires a lower magnetic field. Thus, shield P0.01 is magnetized faster than the others (minimum value of H_max_ = 4.6 Oe), and shield P0 is saturated last (maximum value of H_max_ = 8 Oe). The high saturation magnetic field for shield P0 corresponds to a high SE_max_ value. However, this does not explain the fact that the WWR greatly increases with decreasing grain and magnetic domain size. Probably, the explanation for this is the disordered structure due to the large number of small grains and domains. Within the domain, the magnetization vectors are co-directional, but there is some deviation of the vectors at its boundaries. Within the nanoscale domain, the contribution of boundary disordering increases. There is a possibility of a partial transition to the superparamagnetic state for the P0.01 sample. It is possible that the coexistence of a ferromagnetic and superparamagnetic region in a nanograined shield can significantly increase the WWR of the shield. However, quantitative confirmation of this conclusion requires careful studies of the magnetic properties using SQUID (superconducting quantum interference device) techniques, which are planned to be carried out in the future in the framework of the work not related to the effectiveness of shielding.

## 4. Conclusions

A set of permalloy coatings protecting against a permanent magnetic field was obtained using stationary and pulsed electrodeposition with a variable pulse duration (1, 0.1, and 0.01 s). The shields had a composition of 50 wt % Fe + 50 wt % Ni with small changes of no more than a few percent, which were caused by a change in the kinetics of the redox reaction. XRD studies of the crystal structure have shown that the unit cell parameter and cell volume decrease nonlinearly from 3.578 to 3.567 Å and from 45.80 to 45.38 Å^3^ with transition from stationary to pulsed electrodeposition and with decreasing the pulse duration. The reason for the compression of the crystal lattice can be two factors: the surface compression of grains with a reduction in their size, and the effect of the chemical composition. The microstructure was investigated using SEM and AFM. All coatings had a pronounced grain structure and showed a decrease in the average grain size from 0.5 μm for stationary P0 mode to 100 nm for pulsed P0.01 mode. Along with a decrease in the grain size, the domain size investigated using MFM also decreased. It is shown that samples with 0.3–0.6 µm grains have a disordered domain structure, which makes it possible to achieve higher values of the maximum shielding efficiency (SE_max_ = 29 dB for P0 shield), but these are characterized by a narrow working range. Samples with a grain size of less than 200 nm have an ordered two-domain magnetic structure with a possible partial transition to a superparamagnetic state in regions with a grain size of less than 100 nm. The ordered magnetic structure, the small size of the domain, and the coexistence of ferromagnetic and superparamagnetic regions, although they reduce the maximum value of the efficiency from 29 to 25.5 dB, they significantly expand the operating range of the shields from 24.5 to 33 Oe.

## Figures and Tables

**Table 1:** Technological features of obtaining FeNi shields in direct current (DC) and pulse deposition modes.

Shield Name	Current	Pulse Duration, s	Pause Duration, s	Full Deposition Time, Min	Effective Deposition Time, Min
P0	direct	-	0	85	85
P1	pulsed	1	1	170	85
P0.1	pulsed	0.1	0.1	170	85
P0.01	pulsed	0.01	0.01	170	85

**Table 2:** Crystal structure parameters of FeNi shielding coatings obtained with different electrodeposition modes.

Shielding Coating	CSR, nm	I_(111)_/I_(200)_, %	a, Å	V, Å^3^
P0	3.9	57	3.578	45.80
P1	3.3	84	3.570	45.50
P0.1	5.1	91	3.570	45.50
P0.01	5.0	95	3.567	45.38

## Data Availability

The data of the study is available on reasonable request from corresponding author.
